# Collagen I triggers directional migration, invasion and matrix remodeling of stroma cells in a 3D spheroid model of endometriosis

**DOI:** 10.1038/s41598-021-83645-8

**Published:** 2021-02-18

**Authors:** Anna Stejskalová, Victoria Fincke, Melissa Nowak, Yvonne Schmidt, Katrin Borrmann, Marie-Kristin von Wahlde, Sebastian D. Schäfer, Ludwig Kiesel, Burkhard Greve, Martin Götte

**Affiliations:** 1 Department of Gynecology and Obstetrics, Münster University Hospital, Albert-Schweitzer Campus 1, D11, 48149 Münster, Germany; 2 Department of Radiotherapy-Radiooncology, Münster University Hospital, 48149 Münster, Germany; 3 Present Address: Institut für Molekulare Medizin III, Heinrich-Heine-Universität Düsseldorf, 40225 Düsseldorf, Germany

**Keywords:** Reproductive disorders, Experimental models of disease, Preclinical research

## Abstract

Endometriosis is a painful gynecological condition characterized by ectopic growth of endometrial cells. Little is known about its pathogenesis, which is partially due to a lack of suitable experimental models. Here, we use endometrial stromal (St-T1b), primary endometriotic stromal, epithelial endometriotic (12Z) and co-culture (1:1 St-T1b:12Z) spheroids to mimic the architecture of endometrium, and either collagen I or Matrigel to model ectopic locations. Stromal spheroids, but not single cells, assumed coordinated directional migration followed by matrix remodeling of collagen I on day 5 or 7, resembling ectopic lesions. While generally a higher area fold increase of spheroids occurred on collagen I compared to Matrigel, directional migration was not observed in co-culture or in 12Z cells. The fold increase in area on collagen I was significantly reduced by MMP inhibition in stromal but not 12Z cells. Inhibiting ROCK signalling responsible for actomyosin contraction increased the fold increase of area and metabolic activity compared to untreated controls on Matrigel. The number of protrusions emanating from 12Z spheroids on Matrigel was decreased by microRNA miR-200b and increased by miR-145. This study demonstrates that spheroid assay is a promising pre-clinical tool that can be used to evaluate small molecule drugs and microRNA-based therapeutics for endometriosis.

## Introduction

Endometriosis is a common gynaecological disease in which the uterine lining, the endometrium, grows at ectopic locations such as the ovaries and peritoneal cavity^1^. This disease is currently treated using hormonal therapy and excision surgery. Unfortunately, these treatments are not curative and have high associated side effects and remission rates^1,2^. While targeted therapies are urgently needed, their development has been hindered by the heterogeneity^3,4^ and limited mechanistic understanding of the disease.

**Figure 1:**
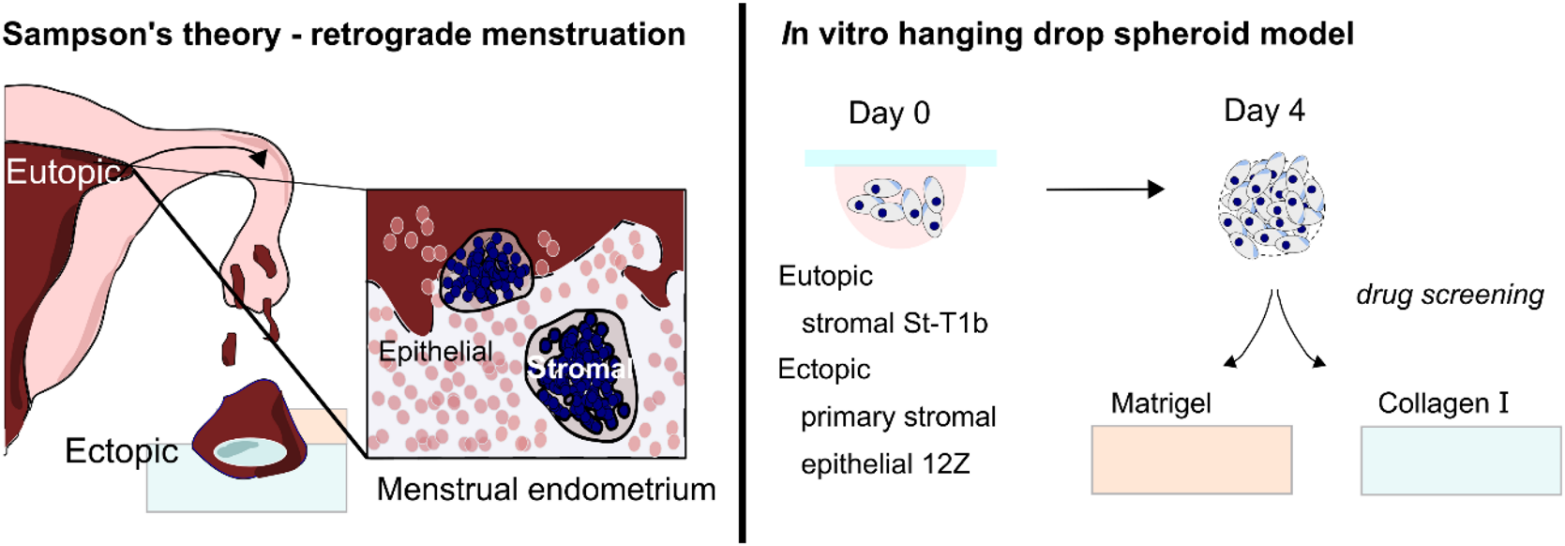
Endometriosis modelling in vitro. Left. Sampson’s theory of retrograde menstruation. Menstrual tissue contains stromal condensates (dark blue) and collapsed epithelial glands (pink). Ectopic lesions are frequently described to have a ‘bullet-like appearance’ Right. Spheroids generated using the hanging drop method as a model of collapsed endometrium architecture are placed on either Matrigel or collagen I on day 4 and their phenotype on the hydrogels and the effect of pharmacological intervention is evaluated.

Based on the widely accepted Sampson’s theory, endometriosis arises when tissue fragments shed during menstruation implant in the surrounding tissue^5^ (Fig. 1). To implant, endometrial fragments have to first penetrate either through intact barriers consisting of epithelial cells, basement membranes and collagen or directly through a damaged tissue (e.g. due to microtrauma^6^ or previous surgical procedure^7–9^) and then spread^10^. In this regard, endometriosis shares many similarities with metastatic cancer^11^. However, while cancer researchers have devoted considerable attention to dissecting the invasive processes involved in cancer metastases^12^, little is known about invasive processes in endometriosis.

One significant hurdle in studying how endometrial cells invade ectopic tissues has been a lack of suitable experimental models^13^. To address this, in vitro models of endometriosis consisting of endometrial explants^14^, organoids^3^ or single cells combined with chorioallantoic membrane^15^, amniotic membrane^16^, peritoneal mesothelial cell monolayers^17^, peritoneal explants^18^ and hydrogels^19^ have been developed. Nevertheless, each of these approaches has some inherent limitations. 2D cell culture is the gold standard, but the invasive and migratory strategies in 2D are markedly different from the coordinated multicellular collective invasion through the extracellular matrix (ECM) that has been observed in vivo^20,21^. Organoid models typically focus predominantly on epithelial cells and lack the stromal component^22^. Explants suffer from high heterogeneity, mixed cell population and low throughput^23^. The promising and integrative tissue engineering approach has so far recreated models of decidual eutopic endometrium rather than lesions or menstrual stage endometrium^19^.

Menstrual stage endometrium is characterized by stromal reorganization into tightly packed cellular condensates sometimes referred to as ‘blue balls’, collapsed glands and blood debris^24^. We hypothesized that such collapsed architecture could be modelled using the spheroid culture in vitro. Spheroid culture is a well-established technique that commonly used to study malignancies^25^. Indeed, endometrial epithelial spheroids generated from 12Z, an endometriotic lesion derived epithelial cell line^26^ and endometriotic stromal cells^27^ have already been shown to share histological similarities to lesions better than 2D culture. However, it has not been investigated how endometrial spheroids interact with the ECM.

In this study, we show that endometrial spheroids create structures resembling lesions on collagen I and Matrigel in vitro within 5–7 days. We demonstrate that this assay can dissect the effect of the cell and ECM type as well as of small molecule- and RNA- drugs.

## Results

### An endometrial stromal cell line (St-T1b), primary endometriotic stromal cells and the endometriotic epithelial cell line (12Z) self-organize into spheroids in hanging drop culture

To capture the heterogeneity of endometrial cells found in lesions, the cells we employed in this study were an immortalized eutopic stromal cell line St-T1b^28^, primary endometriotic stromal cells (ESCs) and the ectopic light red peritoneal lesion derived epithelial 12Z cell line that was previously shown to be invasive in a Matrigel invasion assay^29^.

**Figure 2:**
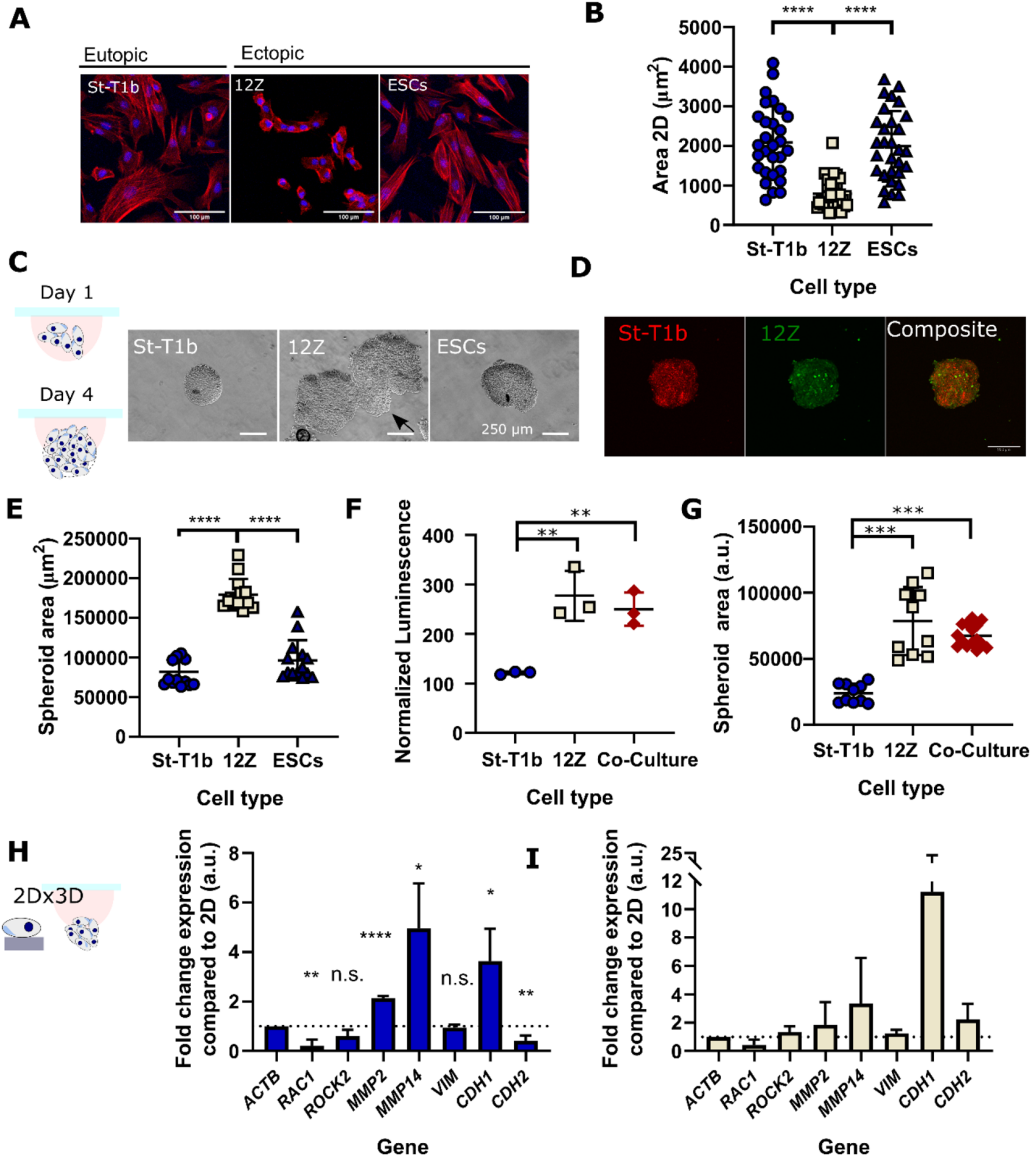
Spheroid formation by endometrial cells. (**A**) F-Actin (red) and nuclei (blue) stained St-T1b, 12Z and ESCs. (**B**) The projected area in 2D of St-T1b and ESCs is significantly larger than of 12Z cells (n = 29, 32 and 30 cells, Kruskal–Wallis with Dunn’s multiple comparisons post hoc test. Data show mean ± s.d.). (**C**) Bright-field images of fixed spheroids that formed after 4-days using the hanging drop method. Scale bars 250 µm. (**D**) Co-Culture 1:1 St-T1b:12Z spheroids on day 4 stained by Cell Tracker. Red are St-T1b and green 12Z cells. Scale bar 200 µm. (**E**) The 12Z spheroids were significantly larger compared to St-T1b and ESCs spheroids (n = 14 prepared across three different preparations, Kruskal–Wallis with Dunn’s multiple comparisons post hoc test), the area was measured manually on bright-field images, 10 × magnification. (**F**) Metabolic-based assay suggests 12Z and Co-Culture spheroids on day 4 consist of a higher number of cells than St-T1b spheroids (n = 3 independent wells and one preparation, one-way ANOVA with Tukey’s multiple comparisons test). (**G**) Spheroid projected area is also significantly larger in 12Z cells and co-culture groups than in the St-T1b group (n = 10–15 independent wells from two different spheroid preparations, Kruskal–Wallis with Dunn’s multiple comparisons post hoc test). (**H**) qPCR analysis comparing gene expression in 2D and 3D spheroids on day 4 of the hanging drop culture in St-T1b cells and (**I**) qPCR analysis comparing gene expression in 2D and 3D spheroids on day 4 of the hanging drop culture in 12Z cells (n = 3 independent preparations on the same cell lines, multiple *t* tests). For all figures in the panel *p < 0.05; **p < 0.01; ***p < 0.001, ****p < 0.0001 and not significant (n.s.) p > 0.05; Data shown as mean ± standard deviation (s.d.) or as mean + s.d.

First, we validated that the cells retained their stromal and epithelial morphology in culture. Figure 2A shows that while the St-T1b and ESCs cells have an elongated, fibroblast-like stromal morphology, 12Z cells have a mostly polygonal shape and grow in clusters. Furthermore, on tissue culture (TC) plastic, the stromal cells exhibit more defined actin fibers compared to the 12Z cells. Quantitative analysis (Fig. 2B) confirmed that 12Z cells are significantly smaller (p < 0.0001) than St-T1b and ESCs, where the average area for St-T1b, 12Z and ESCs cells on TC plastic were 2086 ± 904.1 µm^2^ (n = 29), 787.7 ± 380.9 µm^2^ (n = 32) and 1989 ± 889.5 µm^2^ (n = 30).

Recent studies suggested that spheroid culture offers several advantages over 2D culture and confirmed that 12Z cells^26^ and endometriotic stromal cells^27^ can assemble into spheroids using the U-bottom 96 well plates^27^. However, it has not been investigated whether also the hanging drop method can be used to fabricate endometrial spheroids and whether there are any differences between spheroids fabricated from epithelial and stromal endometrial cells alone or their co-culture. We, therefore, evaluated the hanging-drop method, each drop containing 20,000 of stromal or epithelial cells or their co-culture in 20 µL of standard media and selected day 4 as the harvesting day.

Bright-field images (Fig. 2C) show that all the studied cell types self-organized into spheroids. Interestingly, the morphology of the spheroids varied across cell types. St-T1b and ESCs cells assembled into compact, round-spheroids, while the 12Z spheroids were larger and sometimes exhibited slightly branching morphology. We also generated co-culture spheroids from the epithelial 12Z and stromal St-T1b cell lines combined at 1:1 ratio (Fig. 2D). Cell Tracker staining and confocal imaging suggest the two cell populations were homogeneously distributed throughout the spheroid on day 4. Interestingly, while the size of individual 12Z cells in 2D is significantly smaller compared to the ESCs and St-T1b cells, 12Z spheroids were significantly larger compared to St-T1b and ESCs (n = 14, p < 0.0001 and p < 0.001) (Fig. 2E). To exclude that this is due to a cell-counting error, the spheroid size was measured on spheroids prepared three independent times. The co-culture spheroids were also significantly larger compared to St-T1b spheroids (n = 11) and had a higher metabolic activity that is indicative of higher cell count and proliferation over the spheroid formation period (Fig. 2F,G).

Next, we evaluated whether the condensation into spheroids induces changes in gene expression. We analysed a subset of genes related to ectopic tissue invasion. Gene expression analysis revealed that while organisation into spheroids alters the gene expression of several markers in St-T1b cells, none of these markers was significantly altered in 12Z spheroids compared to monoculture across three independent preparations (Fig. 2H,I).

First, we examined the expression of Ras-related C3 botulinum toxin substrate 1(*RAC1*/Rac1), a small signalling G protein that directs actin-driven cellular protrusion, microtubule prolongation and the formation of lamellipodia^30^ both in single cells and at the leading edge during collective migration^31^. The expression of *RAC1* was significantly downregulated in 3D compared to 2D St-T1b (p < 0.01, n = 3) (Fig. 2H).

Spheroid St-T1b culture exhibited higher proteolytic gene expression compared to 2D (Fig. 2H). qPCR analysis revealed that the spheroids exhibit higher expression of the secreted *MMP2* (p < 0.0001, n = 3) and the membrane-type metalloproteinase *MMP14* (p < 0.05, n = 3) than cells grown in 2D.

As the epithelial to mesenchymal transition (EMT) and mesenchymal to epithelial transition (MET) have been implicated in the progression of the disease, we further investigated the expression of mesenchymal markers vimentin (*VIM*) and cadherin-2 (*CDH2*) and the epithelial marker cadherin-1 (*CDH1*) (Fig. 2H). Vimentin expression remained unchanged in both cell lines (p > 0.05, n = 3). The expression of *CDH2*, a cadherin known to promote invasion in many cell types^32^, was downregulated in St-T1b spheroids (p < 0.01, n = 3) while the expression of *CDH1* was upregulated in St-T1b spheroids (< 0.05 = n = 3) compared to the 2D control.

### Matrigel and collagen I trigger distinct phenotypes in single cells and spheroids where stromal condensates create defects on collagen I

Having confirmed that endometrial stromal and epithelial endometriotic cell line as well as their co-culture were able to form spheroids, we evaluated their invasive behaviour on two different ECM-derived hydrogels: Matrigel and collagen I using confocal imaging.

**Figure 3:**
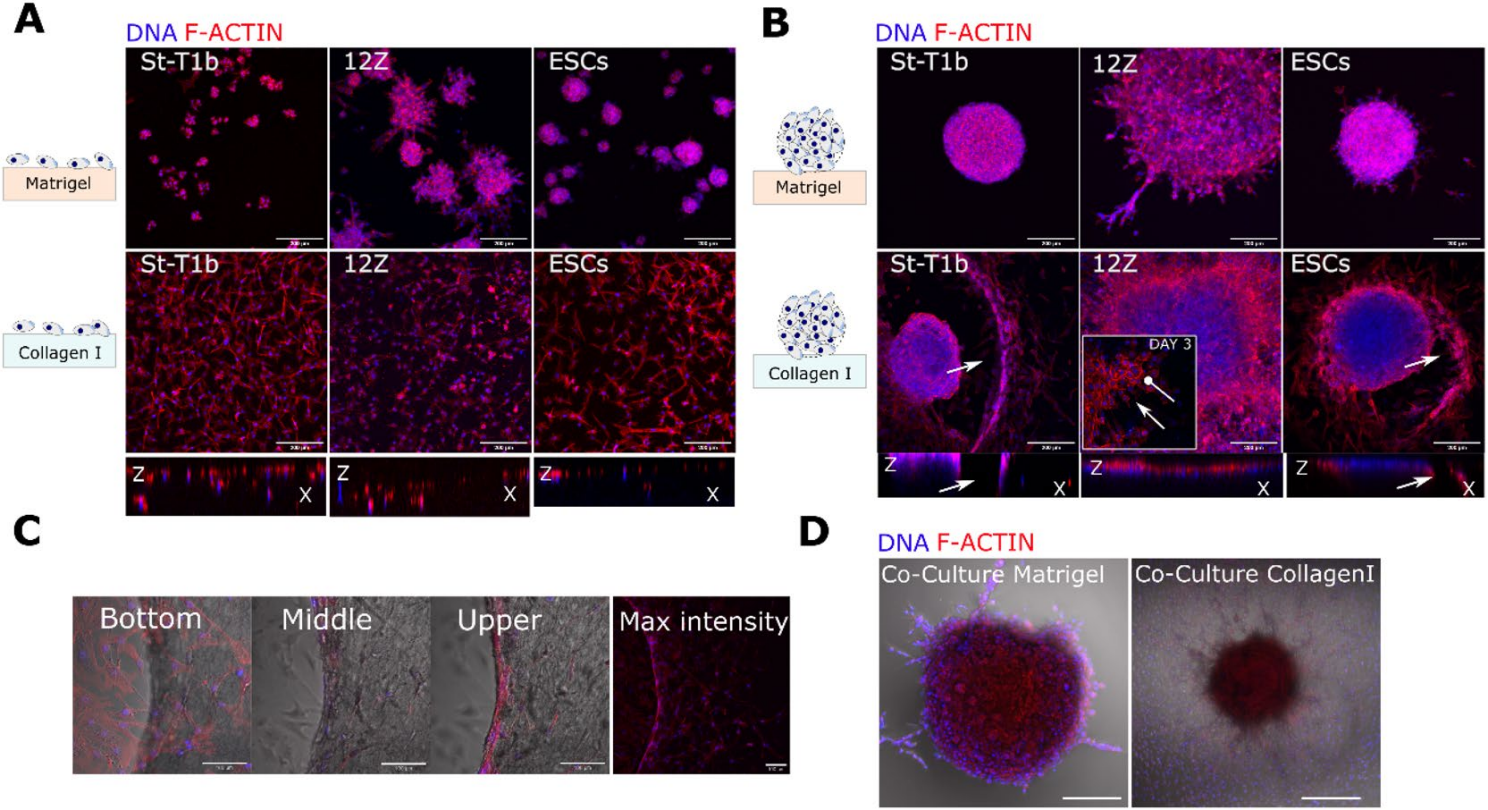
Lesion-like structures on collagen I and Matrigel. (**A**) Confocal images of a suspension of endometrial cells after 3 days on Matrigel (top row). Stromal St-T1b and ESCs cellular aggregates consisted of only a few cells and were highly circular and 12Z aggregates were larger and showed protrusions. All cell types invaded collagen I (bottom row) as single cells (maximal intensity projection, scale bar 200 µm, f-actin red, nuclei blue). (**B**) Confocal images of spheroids after 7 days on Matrigel and collagen I. 12Z exhibited the highest number of protrusions on Matrigel. St-T1b and ESCs created circular defects in collagen I surrounded by cells, whereas epithelial 12Z cells migrated as a sheet and confocal imaging revealed no invasion (maximal intensity projection, scale bar, 200 µm, actin cytoskeleton red, nuclei blue). (**C**) Detail of three different imaging planes of the edge of the circular defect in St-T1b spheroid in collagen I group. F-actin in red and DNA in blue. Scale bar 100 µm. (**D**) S-T1b: 12Z co-culture after 7 days on Matrigel (left) and collagen I (right). Scale bar 200 µm.

Single cells of all studied cells on Matrigel formed cellular aggregates by day 3(Fig. 3A). While these aggregates remained mostly rounded in St-T1b and ESCs groups, the 12Z cell line aggregates consistently developed multiple multicellular protrusions across several preparations. Cells seeded on collagen I were invading collagen I as single cells (Fig. 3A).

We next evaluated the spheroid behaviour on Matrigel and collagen I. On the basement membrane (BM) mimic Matrigel, the stromal St-T1b spheroids remained rounded with ESCs exhibiting few protrusions and only the 12Z spheroids consistently developed multiple multicellular protrusions across several preparations. Confocal imaging on day 7(Fig. 3B) revealed that the 12Z protrusive edges consisted of tightly packed cells (DNA in blue) with scant cytoplasm (actin staining in red).

The response of all studied cell types to collagen I as spheroids was markedly different compared to single cells (Fig. 3B). St-T1b and ESC spheroids on collagen I developed into invasive lesion-like structures (Fig. 3B). More specifically, the St-T1b and ESC spheroids gradually invaded collagen I, leaving behind a circularly remodeled matrix with a ring of tightly adhering cells at its margins (Fig. 3B,C). These rings appeared to stabilize the defect and to limit further random cellular spreading outside of the defect in many but not all spheroids.

Interestingly, no matrix defect or directional spreading was detected in co-culture St-T1b:12Z spheroids on collagen I (Fig. 3D). Co-culture spheroids on Matrigel developed protrusive edges similar to the 12Z-only spheroids.

### Directional invasion followed by the formation of a circular defect occurs in St-T1b and ESCs spheroids but not in St-T1b:12Z co-culture

**Figure 4:**
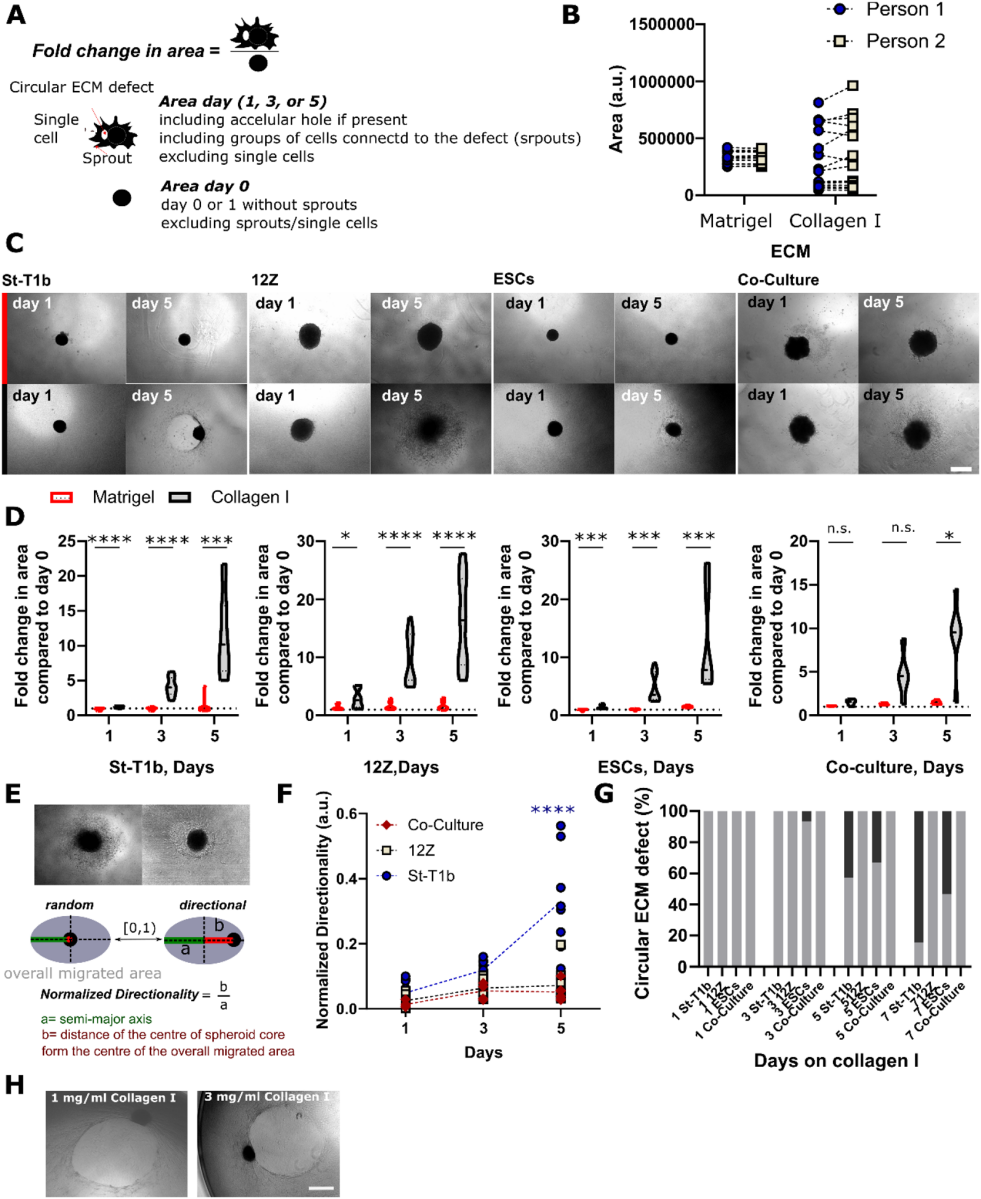
Quantification of spheroid behaviour on Matrigel and collagen I. (**A**) Schematic illustrating how the ‘Fold increase in the area’ was measured and calculated. (**B**) Validation of the manual measurement method showed no significant difference between different assessors following the defined criteria (n matrigel = 13, n collagen = 16, *t* test for each condition). (**C**) Brightfield images on day 1 and 5 of the spheroids on Matrigel (top row) and collagen I (bottom row). Scale bar 500 µm. (**D**) Quantification of the fold change in area for individual cell types and St-T1b:12Z co-culture (n = 12–15 independent wells per time point and condition collated from three independent spheroid preparations, n_cocultures_ = 4–5, one preparation. Two-way Repeated Measures (RM) ANOVA, Šidák’s multiple comparisons tests). (**E**) Schematic illustrating how normalized directionality was calculated. (**F**) Normalized directionally for St-T1b (circles), 12Z (squares) and co-cultures (diamonds) on day 1, 3 and 5 (n = 5–10 wells per experiment collated from two independent preparations, n _co-culture_ = 5 from one preparation). (**G**) Circular ECM defect quantification-grey colour represents the absence of macroscopic ECM defect and the black colour a presence of a circular defect (n = 13–15 independent wells collated from three separate spheroid preparations, n_co-culture_ = 5 from one preparation. Two-way Repeated Measures (RM) ANOVA, Šidák’s multiple comparisons test) (H) Directional matrix remodeling resulting in a circular defect occured on both 1 mg/mL and 3 mg/mL collagen I hydrogels. For all figures in this panel *p < 0.05; **p < 0.01; ***p < 0.001, ****p < 0.0001, and n.s. p > 0.05.

Next, we quantified the invasive and migratory patterns on Matrigel and collagen I using bright-field imaging and a parameter that we termed ‘Fold change in the area’ that we defined as the overall projected area, including matrix defects on the day of interest divided by the area on the day 0 or 1 without any sprouts (Fig. 4A). All analysis was done manually in FIJI using the freehand selection tool. While manual measurement has its limitation, especially when the ‘Area’ increases and its margins become irregular, no significant difference in measured areas was observed between different assessors (Fig. 4B).

Our data show that the ‘Fold change in area’ is significantly higher on collagen I compared to Matrigel across all studied cell types by day 5 (Fig. 4C,D). Confocal imaging combined with brightfield microscopy suggested the stromal spheroids invade (Fig. 3B,C) and migrate on the collagen I matrix directionally (Fig. 4C). To quantify this, we used the parameter ‘Directionality’ that is calculated as the ratio of the distance of the centre of the spheroid core from the centre of the overall migrated area *b* to the semi-major axis of the overall migrated area *a* (Fig. 4E). The normalized directionality increased for St-T1b but not for 12Z or co-culture spheroids with time on collagen I, especially between days 3 and 5 (Fig. 4F). The directional invasion was typically followed by matrix remodeling resulting in a circular defect at the area with the densest stromal cell population (Fig. 4G). In our system (3 mg/mL, 40 µL/well) this typically occurred around day 5 or 7 with 84.6% and 53.3% of St-T1b and ESCs, respectively, having a defect on day 7 (n = 13–15 per time point) (Fig. 4G). The defects formed both on 1 mg/mL and 3 mg/mL collagen I hydrogels, suggesting this behavior occurs across a range of collagen I concentrations (Fig. 4H).

### Spheroid 3D culture as an effective tool to screen small molecule drug and microRNA-based therapeutics

We then evaluated the potential of the here presented endometrial spheroid in vitro assay to screen the potential therapeutic effect of mechanoregulatory small molecules and micro RNAs (Supplementary Table ST1).

### The broad-spectrum MMP inhibitor NNGH limits the invasive behaviour of stromal spheroids on collagen I

**Figure 5:**
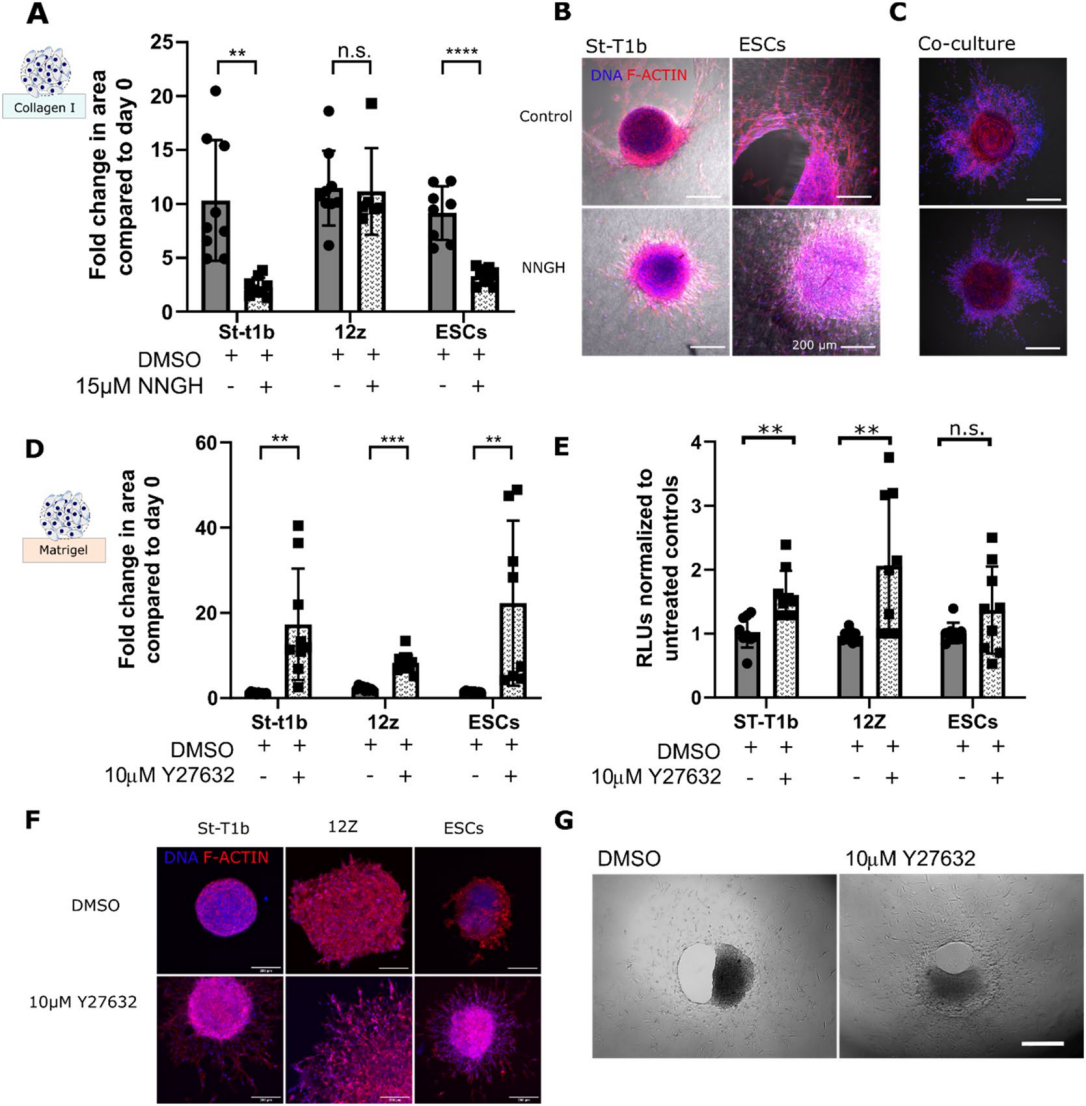
The effects of small molecule inhibitors on lesion formation. (**A**) The broad spectrum MMP inhibitor NNGH significantly reduced the in vitro lesion size in St-T1b and ESCs but not in 12Z cells that migrated on collagen I surface. The spheroid size was measured manually on days 0 and 5 (n = 6–9 independent wells across two preparations, multiple *t* tests). (**B**) NNGH effectively prevented stromal cells from degrading collagen I (bright field channel) but did not completely prevent the cells from migrating. Confocal images were obtained on fixed samples after 7 days in culture. Scale bar, 200 µm. (**C**) Co-cultures on collagen I without (top) and with (bottom) NNGH inhibitor on day 5 Scale bar, 200 µm. (**D**) The ROCK inhibitor Y27632 significantly increased the spreading of endometrial cells on Matrigel after 5 days. Data were compared to the spheroid size on day 0 using bright-field images (n = 8–10 independent wells across two different spheroid preparations, multiple *t* tests). (**E**) Y27632 significantly increases metabolic activity in all studied cell types on day 5 (n = 8–9, multiple *t* tests, three independent preparations). (**F**) Confocal images demonstrating the increase in the projected area of spheroids of all cell types on Matrigel upon Y27632 treatment Scale bar, 200 µm. (**G**) ESCs on Collagen I on day 7 with and without Y27632. Y27632 did not prevent collagen I remodeling. Scale bar 500 µm *p < 0.05; **p < 0.01; ***p < 0.001 and n.s. p > 0.05; Data shown as mean ± s.d.

Previous studies implicated that MMP signalling plays a role in the formation of early endometriotic lesions^15^. Our study shows that the broad-spectrum MMP inhibitor 15 µM *N*-isobutyl-*N*-(4-methoxyphenylsulfonyl) glycyl hydroxamic acid (NNGH) significantly reduced ‘Fold change in the area’ on collagen I from 10.4 fold to 2.3 fold (n = 6–9) and 9.2 fold to 3.3 fold (n = 6–9) in St-T1b and ESCs, respectively, but did not significantly affect the ‘Fold change in the area’ in 12Z cells (n = 6–9) (Fig. 5A, Supplementary Figure S1). Furthermore, it can be seen from Fig. 5B, that while NNGH treatment prevents the formation of the circular defect on collagen I even after 7 days in culture, the migration of St-T1b and ESCs is not completely eliminated. The effect of NNGH inhibitor on the St-T1b:12Z co-culture was less pronounced and neither the control nor NNGH group formed matrix defects by day 5 (Fig. 5C).

### ROCK inhibition significantly enhances spreading and invasion of all studied endometrial cell types on Matrigel

The ROCK inhibitor Y27632 significantly (p < 0.01) increased the ‘Fold change in the area’ of all studied cell types on Matrigel compared to DMSO (Fig. 5D). The area occupied by St-T1b, 12Z and ESCs was 17.3, 6.6 and 22.3 fold larger compared to day 0 (Fig. 5E). Y27632 also affected the numbers of metabolically active cells, which were significantly higher compared to controls for St-T1b and 12Z cells on day 5 on Matrigel. Moreover, Y27632 affected spheroid morphology (Fig. 5F). Y27632 on Collagen I resulted in a disaggregation of the spheroid core in St-T1b and ESCs as shown in the Supplementary Figure S2. Treatment with Y27632, in contrast to the MMP inhibitor NNGH, did not prevent Collagen I matrix remodeling in ESCs (Fig. 5G) suggesting the directional remodeling is rather due to proteolytic action than acto-myosin contraction.

### The spheroid model reveals context-dependent roles of the mechanoregulatory microRNAs miR-200b and miR-145 on the invasive behaviour of the endometriotic epithelial cell line 12Z on Matrigel

**Figure 6:**
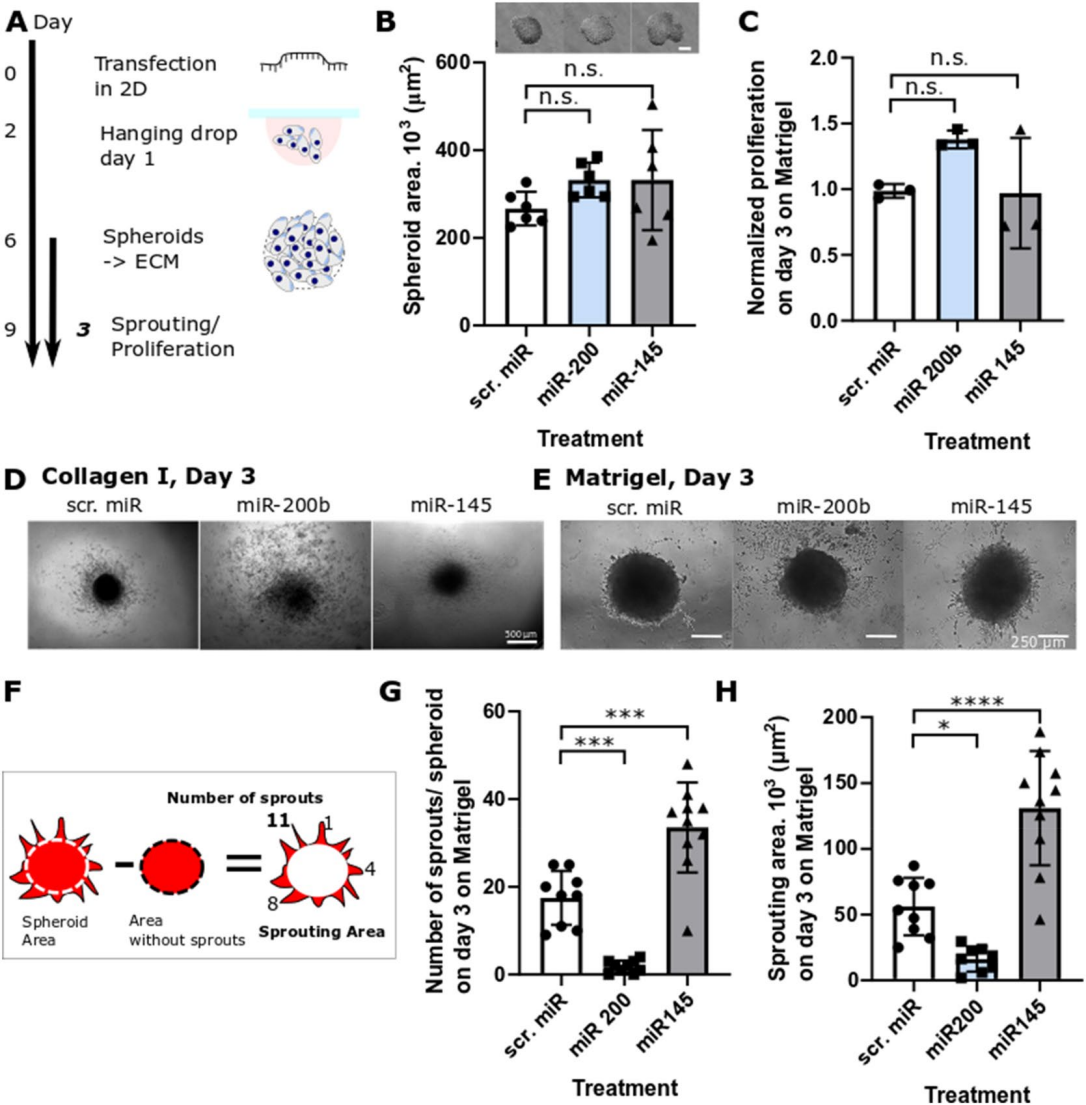
The effect of microRNA on 12Z sprouting on Matrigel. (**A**) Schematic of the workflow (**B**) none of the microRNAs affected the ability of 12Z cells to self-organize into spheroids and all groups resulted in spheroids with similar area (scale bar = 250 µm, n = 6 independent spheroids prepared across two preparations, ANOVA). (**C**) None of the microRNA affected overall metabolic activity measured as luminescence compared to scr.miR treated controls. Data are normalized to controls without any microRNA (n = 3 independent wells, ANOVA, one repeat). (**D**) Representative images of microRNA treated 12Z spheroids after 3 days on collagen I. Scale bar, 500 µm. (**E**) Representative images of microRNA treated 12Z spheroids after 3 days on Matrigel. Scale bar, 250 µm. (**F**) A diagram showing how the number of sprouts and the sprouting area parameters were calculated. (**G**) miR-200 significantly decreased while miR-145 significantly increased the number of sprouts per spheroid after 3 days on Matrigel. (n = 9–10 independent wells across two independent preparations, ANOVA, Tukey’s multiple comparisons). (**H**) The overall area occupied by sprouts was significantly larger and smaller when treated with miR-145 and miR-200b, respectively, compared to scr.miR after 3 days on Matrigel (n = 8–10 independent wells across two different preparations, ANOVA, Tukey’s multiple comparisons, two independent experiments), *p < 0.05; **p < 0.01; ***p < 0.001 and n.s. p > 0.05; data expressed as mean ± s.d.

We next investigated whether our in vitro model can be used as a tool to screen the functional effect of various microRNAs on endometrial phenotype. In particular, we selected two microRNAs, miR-200b^33^ and miR-145^34^, that have been previously shown to be dysregulated in endometriosis^35^ and to modulate the invasion and migration of 12Z cells in 2D and Transwell assays. miR-200b acts as a transcriptional repressor of ZEB1/2 and thus downregulates EMT transition^36^. The miR-145 is upregulated in endometrial lesions and has been described to modulate cytoskeletal dynamics in several cell types, including endometrial, and has many validated targets, including beta and gamma actin, cofilin, fascin, myosin light chain 9 and Rho kinase Rock1^34,37^. The transfection was performed in monolayer culture before the fabrication of spheroids and the effect of microRNAs on spheroid spreading was assessed after 3 days on Matrigel to minimize the effect of miR dilution and degradation^38^ (Fig. 6A). It can be seen from Fig. 6B that microRNA transfection did not significantly alter the ability of cells to form spheroids and the area of individual spheroids was not significantly different (p > 0.05) across the treatment groups nor was the proliferation (Fig. 6C). We observed spheroid fragmentation of miR-200b transfected cells on Collagen I which resulted in a discontinuous nature of the projected area the size of which could not be reliably quantified (Fig. 6D). MiR-145 significantly reduced the spheroid area compared to scr. miR controls on day 3 on collagen I (Supplementary Figure S3). On Matrigel, the microRNAs, affected sprouting characteristics behaviour of 12Z cells as seen in the bright-field images in Fig. 6E. The miR-200b treatment significantly decreased the number of sprouts per spheroid from ~ 17 to ~ 1, while miR-145 significantly increased the number of sprouts per spheroid to ~ 34 (Fig. 6F,G) and increased the overall sprouting area from 56.12 × 10^3^ ± 21.87 × 10^3^ µm^2^ per scrambled control miR spheroid to 130.86 × 10^3^ ± 43.47 × 10^3^ µm^2^ per miR-145 treated spheroids (p < 0.0001) (Fig. 6F,H). In line with previous findings on EMT-marker analysis in 2D-cultured 12Z cells^31,33^, qPCR analysis of miR-200b-treated 12Z spheroids indicated strong upregulation of CDH1 expression levels, however, the data were not significant due to high variability, since only minute amounts of RNA could be isolated from the spheroids (Supplementary Figure S4).

## Discussion

Endometriosis is a complex multifactorial disease^1^. The overall goal of this study was, therefore, to develop a modular 3D in vitro model that makes it possible to study the interplay of different factors that have been proposed to contribute to the pathogenesis of endometriosis and screen potential therapeutics in vitro.

First, we demonstrate that the hanging drop method makes it possible to generate endometrial spheroids of reproducible size and thus provides a good alternative to the low-adhesion plate method^26,39^. Our data show that the spheroid size is consistently cell-type specific, with stromal cells generating smaller spheroids than the epithelial 12Z cells or their co-culture. This is likely due to proliferation of 12Z cell in spheroids as suggested by the cell proliferation assay on spheroids on day 4. qPCR analysis revealed that the spheroid culture affects gene expression. The stromal St-T1b had enhanced expression of the MMP2, MMP14 compared to 2D culture. RAC1, on the other hand, was downregulated in St-T1b spheroids. Spheroids in which Rac1 production was either inhibited or the gene was constitutively expressed had suppressed or enhanced migration in 3D matrices, respectively^40^. We speculate that it is possible that RAC1 is temporarily downregulated in stromal cells cultured as a suspension spheroid culture. While basal CDH1 expression was very low with a Ct value of 27, as expected for a mesenchymal cell line, we observed a significantly increased expression in 3D culture. We could previously show that CDH1 mRNA expression can be induced in St-T1b cells by external stimuli such as seminal plasma^41^, however, the upregulation in a 3D environment warrants further investigation. While 12Z cells have been initially characterized as being CDH1 negative^28^, low expression levels have been subsequently detected by our group in cells authenticated by STR analysis^32,33^. Our experiments suggest there is no significant difference in any of the analysed markers CDH1^33^, RAC1, ROCK, MMP2 and MMP14 in the 12Z cell line. While another study showed MMP2 expression is upregulated in spheroid compared 2D culture in 12Z cells^26^, this difference could be due to different spheroid size and culture time.

Endometriosis is marked by the growth of endometrium at ectopic locations^1^. We, therefore, investigated how epithelial 12Z, ESCs and St-T1b and co-culture spheroids; and single cells interact with two ectopic ECM mimics Matrigel resembling the basement membrane and collagen I mimicking the exposed stroma. The ‘fold increase in the area’ of the spheroids was markedly higher on collagen I than on Matrigel on day 5. Similarly, single cells seeded on top of these hydrogels preferentially invaded collagen I hydrogels. Our results are in agreement with previous studies conducted on cancer cells suggesting that collagen I alone can increase the invasive cellular phenotype and show that this effect is significant across cell types^42–44^. These data also tie well with the previously reported clinical observations that tissue scarring either due to surgery or persistent microtrauma could contribute to the pathogenesis of endometriosis^7–9^.

While the 12Z cell line was created from lesion-derived cells^29^ based on their ability to penetrate through Matrigel coated invasion chambers, the 12Z single cells in our study only assembled into cellular aggregates with processes and 12Z spheroids developed invasive edges. Previously, Pollock and colleagues also observed only low levels of basal invasion in 12Z cells on Matrigel hydrogels^45^. We speculate that the limited invasive capacity of 12Z cell observed in this study could be due to the chemotactic gradient that is a key part of the invasion chamber setup. Our group has indeed previously demonstrated that 12Z are invasive under a fetal calf serum gradient^33^.

Unexpectedly, there was a marked difference between the behaviour of stromal single-cell suspension and spheroids on collagen I. The St-T1b and ESC spheroids but not single cells consistently migrated on, invaded and remodelled collagen I in a directional manner leaving behind a circular defect in the material encircled by the cells that visually resembled peritoneal endometriotic lesions. Given that this was the case for both the St-T1b cell line derived from healthy cells and ectopic ESCs suggests such invasive behavior might be an inherent property of stromal endometrial menstrual condensates and could be critical not only for the pathophysiology of endometriosis but also for normal regeneration of endometrium.

Directional migration followed by matrix remodeling was not observed in the 12Z-spheroid or the 12Z: St-T1b co-culture groups, suggesting the stromal-epithelial interactions modulate stromal invasiveness. While we did not investigate the MMP levels of the co-culture spheroids, previous research determined that the co-culture between endometrial Ishikawa epithelial and telomerase-immortalized stromal cells reduces the MMP2 levels in stromal cells both in the absence of hormonal stimulation and in the presence of 10 nM estradiol concentration^46^.

**Figure 7:**
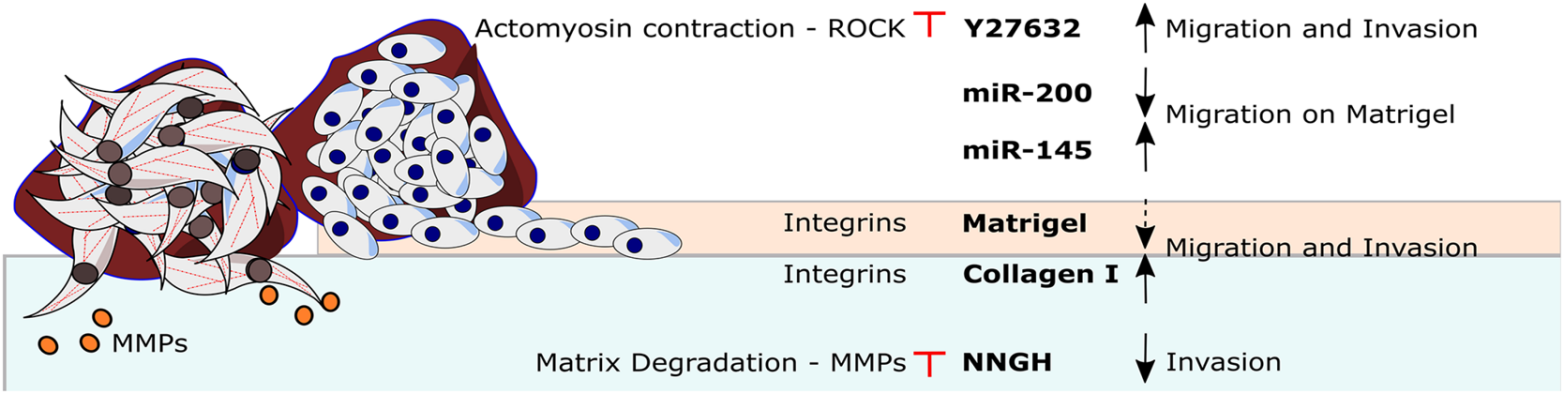
The invasiveness of endometrial spheroids depends both on the cell type (stromal St-T1b and ESCs in brown, epithelial 12Z in blue) and ECM. Invasion and spreading is strongly enhanced by exposed collagen I. Migration on Matrigel is modulated by microRNAs and ROCK inhibitor increases invasion and migration on this substrate. Invasion on collagen is MMP-dependent. Red marks signify experimental intervention. Blunt arrow signifies that an inhibitor was added. Black arrows show the effect of the intervention. Dashed arrow suggest weak effect.

In this paper, we further demonstrate that the endometrial spheroid-ECM platform can be used for drug screening of small molecule drugs and micro-RNAs (Fig. 7). We show that the collagen I circular defect caused by stromal cells arises due to matrix degradation via MMPs rather than due to cellular contraction. Both eutopic and ectopic stromal cells had significantly upregulated MMP expression and the MMP inhibitor, NNGH, significantly reduced the size of in vitro stromal lesions on collagen I. These results are in good agreement with Nap and colleagues that demonstrated that inhibiting MMP activity prevents the development of endometriotic lesions in a model combining chicken chorioallantoic membrane model and biopsies of menstrual stage endometrium obtained from healthy donors^15^. Our results refine this model and show that while, in agreement with the previous studies^47^, the MMP inhibitor significantly slows down the invasion of spreading of stromal cells on collagen it has little effect on the collective migration of 12Z cells. Another signaling molecule we targeted is ROCK, which is a key regulator of the cytoskeleton^30,48^. On Collagen I, ROCK inhibitor Y27632 treatment led to a rapid loss of the spheroid core structure compared to controls and Y27632 did not prevent Collagen I remodeling by ESCs suggesting the matrix remodeling is not primarily driven by matrix contraction but rather by MMP proteolytic action. Y27632 further significantly increased the ‘fold change in area’ and cell numbers in vitro on Matrigel in all studied cell types. Similar increase in cellular spreading following the treatment with ROCK inhibitors have been described in microvascular endothelial cells^49^, retinal pigment epithelial cells^50^ and osteoblastic cells^51^. It needs to be noted that Y27632 has a complex effect on phenotype^52,53^. For example, prior studies demonstrated that Y27632 reduces endometriosis associated fibrosis in vitro^54^.

Another promising class of therapeutics targets microRNA signaling^35,55^. Given that a typical micro-RNA has tens of targets, sequencing studies need to be accompanied by reliable functional assays to be biologically meaningful^56^. In this study, we demonstrate that the spheroid assay can be used to reproducibly evaluate the effect of individual microRNAs on the complex, multicellular spreading of endometriosis-mimicking constructs over several days. We show that miR-200b treatment of 12Z cells resulted in a reduction of sprout formation, which may be indicative of a less invasive phenotype. Our previous 2D data suggest that miR-200b may have reverted the 12Z phenotype to an epithelial-state^33^, however, the paucity of RNA in the spheroids did not allow us to unequivocally confirm this hypothesis, as we saw only a non-significant increase in expression of the epithelial marker E-cadherin (Supplementary Figure S4). Our spheroid model further revealed that while miR-145 reduces the migrated area on Collagen I compared to controls (Supplementary Figure S3), results which are in agreement with previous in vitro 2D assays^34^, the microRNA miR-145 up-regulated in ectopic lesions in vivo increases 12Z sprouting on Matrigel in vitro. These findings were unexpected and investigating this into more detail is beyond the main focus of this study. Nevertheless, there is an increasing appreciation that cells adopt a host of invasive and migratory strategies that are highly context-dependent and enabled by distinct signaling pathways. Liu and colleagues observed that miR-145 upregulation enhances angiogenesis, including the sprouting from aortic rings and linked this to the suppression of tropomodulin 3 (TMOD3)^57^ while we observed that miR-145 inhibits proliferation and migration in breast cancer and endometriotic cells using the Transwell migration and scratch assays^32,58^. Therefore, miR-145 might influence cellular invasive behaviour not only in cell-type but also invasive/migratory-mode manner and ECM-substrate-dependent manner. While the majority of oncological studies on miR-145 function suggest that it reduces invasive growth by targeting a variety of mRNAs, two studies in trophoblast cells have described invasion-promoting functions of miR-145, which were attributed to a targeting of mucin 1 (MUC1) and leukemia inhibitory factor receptor (LIFR), respectively^59,60^. We can only speculate that the 3D spheroid culture compared to 2D culture of 12Z cells may have altered the expression patterns of miR-145 target mRNAs in a way that alters the response to this epigenetic regulator. For example, miR-145 may target new mRNAs that are not expressed in the 2D setting (or vice versa), resulting in a different net response. Overall, we demonstrate that the spheroid assay can be used as an additional assay to screen for both small molecule and RNA-based therapeutics.

A major limitation of our study is that it relies on cell lines that have been transformed and represent only a limited subset of disease phenotypes and a more extensive primary cell pool will be required to confirm and fully elucidate the here reported findings. We also did not investigate the influence of decidualization. Notably, our study did not incorporate primary endometrial epithelial cells with purely epithelial characteristics. Additionally, the wider implementation of this assay for the study of endometriosis will rely on future advances in the molecular characterization of spheroids and high-throughput image analysis. Furthermore, automated image analysis would significantly increase the throughput of this assay. In recent years, the quality of image processing algorithms has approached that of trained humans while significantly decreasing the time needed to evaluate individual samples^61^. It needs to be noted that for such algorithms either large training datasets or pre-defined criteria are needed. Given the wide array of spheroid phenotypical responses, we have only started to identify such criteria.

Overall, our screening platform provides evidence that the physiological condensation of endometrial stromal cells into spheroids might play an important role in the development of a subset of endometriotic lesions. As such a directional invasive phenotype in vitro is unlikely to arise by chance, endometrial stromal condensation might also have currently unknown but likely important biological role in the cyclical regeneration of normal endometrium. At the same time, our results show that the epithelial lesion-derived 12Z spheroids also rapidly migrate on collagen I and stromal-epithelial interactions modulate the invasiveness of stromal cells. Previous studies indeed revealed significant heterogeneity and variability among different endometriosis subtypes with several sub-types staining predominantly for stromal markers^62^.

In conclusion, this study documents that endometrial stromal cell line St-T1b and primary endometriotic stromal cells engage in directional migration with significant collagen I remodeling when cultured in spheroid culture and that this behaviour is inhibited by the broad-spectrum MMP inhibitor NNGH. We anticipate that this assay will be used to gain further insights into invasive processes involved in endometriosis and for the screening of both small molecule and RNA-based drug candidates and their off-target effects.

## Methods

### Cell culture

The 12Z ectopic epithelial cell line^17,29^ was maintained in DMEM media (Sigma-Aldrich, cat. No. D0819, Deisenhofen, Germany,) supplemented with 10% FBS (Biochrom GmbH, cat. no. S0615, Berlin, Germany) and 1% Pen/Strep (Sigma-Aldrich, cat. No. P4333). The St-T1b cell line^28^ and primary ectopic lesion-derived stromal cells (ESCs) were maintained in 70% DMEM/18% MCDB 105 media (Sigma-Aldrich, cat. No. 117-500) supplemented with 10% FBS, 1% Pen/Strep, 1% Glutamine and 5 µg/mL insulin (Sigma-Aldrich, cat. No. 10516). Cells were routinely split twice a week. ESCs were prepared from ectopic lesions and characterized as previously described^63^. Primary endometriotic stromal cells were prepared from a biopsy of a woman with endometriosis who underwent surgical treatment at the Department of Gynecology and Obstetrics of Münster University Hospital in 2013, and stored as aliquoted stocks in liquid nitrogen, which were freshly thawed and passaged in routine culture two times prior to usage in the experiments described. The modified American Society for Reproductive Medicine classification was used to assess endometriosis^64^. For all ESC experiments, stroma cells derived from a lesion located at the pelvic wall (rASRM score II) of a 19-year-old patient were employed. The study was carried out following the Declaration of Helsinki and approved by the local ethics commission (Ethikkommission der Ärztekammer Westfalen‐Lippe und der Medizinischen Fakultät der WWU; approval no. 1 IX Greb 1 from 19 September 2001, updated 2012). The participant gave written informed consent.

### Spheroid formation

Spheroids were generated using the hanging drop method^65^, where 20 µL drops each containing 20,000 cells were deposited on the top lid of a plastic Petri dish and the bottom chamber was filled with sterile water or PBS (Sigma-Aldrich, cat. No. D1408). The spheroids were harvested after 4 days at 37 °C and 7.5% or 5% CO_2_. The co-culture spheroids were formulated at 1:1 12Z:St-T1b ratio.

### Preparation of collagen I and Matrigel

A 3 mg/mL collagen I hydrogel was formed by neutralizing and diluting the stock solution of Collagen Type I Rat Tail matrix (Corning, Bedford, MA, USA, cat. No. 354236, 4 mg/mL or 3.4. mg/mL batch) with 1 N NaOH (Applichem, cat. No. A1432, Darmstadt, Germany), 10 × PBS (Sigma-Aldrich, cat. No. D1408) and chilled deionized water. The amount of 1 N NaOH was calculated as 1 N NaOH volume = (volume of the stock collagen) × 0.023 mL. The amount of 10 × PBS was calculated as volume 10 × PBS = (final volume)/10. Phenol red-free Basement Membrane Matrix Growth Factor Reduced Matrigel (Corning, cat. No. 356231) was thawed on ice prior to use. The gels were deposited into pre-chilled 96-wells at 35–40 µL per well in 9.2–9.4 mg/mL Matrigel. Each 96-well plate was subsequently sealed with parafilm and the gels were left to solidify for 30–60 min at 37 °C. For higher magnification confocal imaging, collagen and Matrigel were deposited on glass coverslips.

### Spheroid response to collagen I/Matrigel

Following gel formation, the wells in a 96-well plate were filled with 50 µL of phenol-red free DMEM (Gibco, cat. No. 21063-029, Darmstadt, Germany) supplemented with 5% charcoal-treated FBS (Biochrom GmbH, cat. no. S0615) and 5 µg/mL insulin solution (Sigma-Aldrich, cat. No. 10516). Subsequently, one to three spheroids per well were manually added to individual wells. The media were changed every 3–5 days and the samples were kept in an incubator at 37 °C and 7.5% CO_2_. The spheroids were imaged on day 1, 3, 5 and 7.

### Metabolic activity measurement

Viability was assessed using the CellTiter-Glo 3D Viability assay (Promega, cat. No. G9681, Walldorf, Germany). Spheroids and surrounding medium were collected after four days and transferred to an opaque-walled 96-well plate. A volume of CellTiter-Glo Reagent equal to the volume of cell culture medium was added. The mix was incubated according to manufacturer instructions and luminescence in the form of relative light units (RLUs) was recorded using a CLARIOstar Plus (BMG Labtech, Ortenberg, Germany).

### Inhibitors

The effects of three inhibitors on spheroid spreading were evaluated. The MMP inhibitor NNGH (Merck, cat. No. SML0584, Darmstadt, Germany) was stored at 15 mM in DMSO and dissolved to the final concentration of 15 µM in media and the ROCK inhibitor Y27632 (Sigma-Aldrich, cat. No. Y0503, 10 mM stock) at 10 µM. In all experiments, spheroids were added directly to inhibitor-containing media. Inhibitor-containing 5% charcoal-treated FBS/insulin media were exchanged every 3 days.

### microRNA transfection

The transfection with negative control microRNA (Scr. miR), miR-200b and miR-145 (Table 1) was performed in a 6-well plate on 60–70% confluent cells. Before transfection, the growth media were exchanged for Opti-MEM I Reduced Serum Media (Gibco, cat. no. 31985-070, Thermo-Scientific, Germany). The transfection with 20 nM microRNA of interest (Table 1) was conducted using the Dharmafect reagent (Dharmacon, cat. no. T-2001-03, Lafayette, CO, USA). The cells were incubated with the transfection mixture for 24 h when the media were exchanged for full growth media. MiR spheroids were fabricated 48 h after the addition of transfection media.

**Table 1:** MicroRNAs used in the study.

MiR	Specifications	Cat. number	Manufacturer
Scr. miR	Pre-miR Negative Control 2	AM17111	Ambion, Darmstadt, Germany
miR-200b	hsa-miR-200b-3p: MC 10492, mirVana, miRNA mimic	4464066	Ambion
miR-145	hsa-miR-145, Pre-miR miRNA Precursor	AM17100	Ambion

### Live cell staining and immunostaining

The F-actin cytoskeleton was visualized using Phalloidin CruzFluor 594 Conjugate (Santa Cruz Biotechnology, cat. No. sc-363795, Santa Cruz, CA, USA) at 1:1000 dilution. The nuclei were visualized using DAPI (Sigma-Aldrich, cat. No. D9564) diluted at 1:50,000. The cells were fixed using 3.7% formaldehyde (Merck, cat. No. 1.04003.1000, Darmstadt, Germany) at 37 °C for 15 min. Following washing with PBS (Sigma-Aldrich, cat. No. D1408), the cells were permeabilized with 0.1% Triton-X (Riedel-de-Haen, cat. No. AG 56029, Seelze, Germany) for 5 min. Hydrogels in a 96-well plate were stained by adding 25 µL of the 1:1000 phalloidin dye and incubated for 1 h at 37 °C. Live cells were stained either with CellTracker Green CMFDA (Thermo Fischer, cat. No. C2925) or CellTracker Red CMTPX (Thermo Fischer, cat. No. C34552) at a concentration of 5 µM according to manufacturer’s instructions prior to mixing two cell types to form a co-culture.

### Imaging

Cells were analysed for morphological and cytoskeletal markers. The bright-field images were obtained using either an Axiovert100 (Carl Zeiss, Jena, Germany) or an inverted microscope (Leica, Wetzlar, Germany) using 5 ×, 10 × and 20 × objectives. Confocal imaging was performed on fixed stained samples in a 96 well plate. Samples were imaged with the Zeiss LSM 880 inverted confocal microscope (10 ×, 0.45 NA) (Carl Zeiss, Jena, Germany) equipped with ZEN 2 software and using 11.04 µm z-stack intervals and sequential scanning (514 nm argon laser, 405 nm diode laser, Bright field). The number of sections was adjusted based on the sample thickness.

### Image analysis

All images were analysed in FIJI^66^. Confocal images are depicted as maximal intensity projections. The spheroid area was measured manually by tracing the spheroids using the freehand tool and measure function on Bright-field images of spheroids on Petri Dishes, glass slides or in a 96-well plate. Fold increase in area was calculated as the spheroid area on a given day divided by spheroid size on day 0 or day 1. If on day 1 any protrusions were present and the spheroid was used as a reference size for the given experiment, the protrusions on day 1 were excluded from the analysis to better reflect the size of the original spheroid core. The parameter directionality was calculated as the ratio between the distance in pixels between the centre of the overall migrated area and the centre of the spheroid, divided by the semi-major axis of the overall migrated area of the spheroid (Fig. 4E). The number of sprouts per image was counted manually and the sprouting area was calculated as the total area occupied by an expanding spheroid with sprouts minus the area occupied by the spheroid without any protrusions (Fig. 6F).

### RNA extraction and cDNA synthesis

mRNA isolation was performed with InnuPREP RNA mini kit (Analytikjena, cat. no. 845-KS-2040250, Jena, Germany) according to the supplier’s protocols. The quantity of RNA was measured on an Eppendorf BioPhotometer (Eppendorf, Hamburg, Germany) and considered pure if the absorbance at 260 nm/280 nm was more than 1.8. The concentration of 0.4 µg RNA/10 µL of dH_2_O was used. cDNA synthesis was performed using High Capacity kit (Applied Biosystems, cat. No. 4368814, Foster City, CA, USA) according to the manufacturer’s instructions on a TGradient thermocycler (Biometra, Göttingen, Germany).

### PCR

Quantitative RT-PCR analysis was performed using 20 ng cDNA per reaction using the Taqman Universal PCR Master Mix (Thermo Fisher, cat. No. 4304437) and SYBR Green PCR Master Mix (Thermo Fisher, cat. No. 4344463). Gene expression values were calculated using the mean C_t_ values of the samples. The expression of target genes was normalized to the housekeeping gene ACT, and then to St-T1b cells line (2^−ΔΔCt^). The primers were synthesized by Biolegio (Nijmegen, The Netherlands) and are listed in Tables 2 and 3.

**Table 2:** Sybr Green PCR primers.

	Forward	Reverse
ACTB	TCAAGATCATTGCTCCTCCTGAG	ACATCTGCTGGAAGGTGGACA
RAC1	CGCCTCCTGTAGTCGCTTTG	CACGCTGTATTCTCGCCAGTG
MMP14	CCATTGGGCATCCAGAAGAGAGC	GGATACCCAATGCCCATTGGCCA
MMP2	GCCGTGTTTGCCATCTGTTT	CTGCAGGGAGCAGAGATTCG
VIM	TCAGCATCACGATGACCTTGAA	CTGCAGAAAGGCACTTGAAAGC
CDH2	TTCTGACAACAGCTTTGCCTCTG	TTTATTCAGAACGCTGGGGTCA
CDH1	CAAAGCCCAGAATCCCCAAG	CACACCTGGAATTGGGCAAA

**Table 3:** PCR primers Taqman.

Actin	hs99999903 m1
ROCK2	hs00153074 m1

### Statistical analysis

Data were analysed using GraphPad Prism8 (GraphPad Software, San Diego, USA). Normal distribution was tested using the Shapiro–Wilk test. A two-tailed unpaired Student’s *t* tests were used to analyse statistical significance between two conditions in an experiment. For experiments with three or more comparisons, an ordinary one-way ANOVA with a Tukey’s multiple comparisons test was used. For data that were not normally distributed, the Kruskal–Wallis test followed by Dunn’s multiple comparisons test was used. A two-way repeated-measures (RM) ANOVA with Šidák’s multiple comparisons test was used to evaluate the effect of Matrigel and collagen I on spheroid size over time. Significance values were chosen as *p < 0.05; **p < 0.01; ***p < 0.001, ****p < 0.0001. Error bars represent the mean ± s.d or mean + s.d. All figure panels were assembled in Inkscape 0.92.

## Supplementary Information

Supplementary Information.
