# Supplementary material for: Collagen I triggers directional migration, invasion and matrix remodeling of stroma cells in a 3D spheroid model of endometriosis
Source: Sci Rep. 2021 Feb 18;11:4115. doi: 10.1038/s41598-021-83645-8 (PMC7892880; doi:10.1038/s41598-021-83645-8)
Supplement: Supplementary file 1 — Supplementary Information. [file 41598_2021_83645_MOESM1_ESM.docx]

**Supplementary information**

**Collagen I triggers directional migration, invasion and matrix remodeling of stroma cells in a 3D spheroid model of endometriosis**

Anna Stejskalova ^1^ **^*^**, Victoria Fincke ^1^, Melissa Nowak ^1,+^, Yvonne Schmidt ^1^, Katrin Borrmann ^2^, Marie-Kristin von Wahlde ^1^, Sebastian D. Schäfer ^1^, Ludwig Kiesel ^1^, Burkhard Greve ^2^, Martin Götte ^1, *^

1 Department of Gynecology and Obstetrics, and 2 Radiotherapy - Radiooncology, Münster University Hospital, D-48149 Münster, Germany

* Address correspondence to Prof. Dr Martin Götte, Department of Gynecology and Obstetrics, Albert-Schweitzer Campus 1, D11, 48149 Münster, Germany, e-mail: [mgotte@uni-muenster.de](mailto:mgotte@uni-muenster.de) or to Dr Anna Stejskalova, e-mail: [anna.stejskalova@gmail.com](mailto:anna.stejskalova@gmail.com)

+ present address: Institut für Molekulare Medizin III, Heinrich-Heine-Universität Düsseldorf, D-40225 Düsseldorf, Germany

**Figure S1**

**
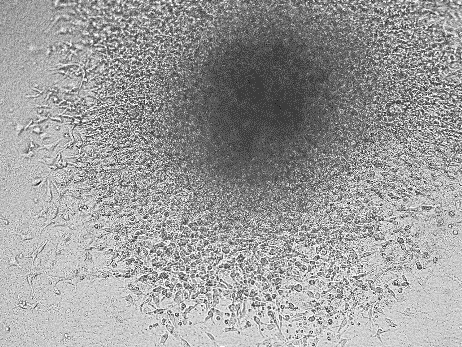

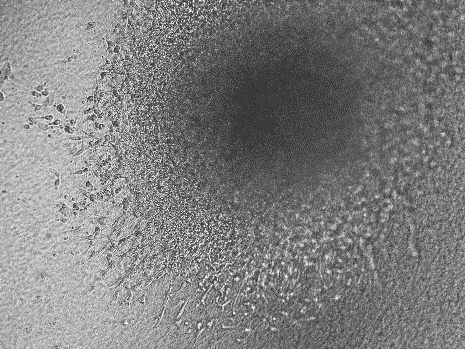
**

Figure S1. Evaluating the effect of small molecule inhibitors. 12Z spheroids controls (left) and treated with NNGH inhibitor (right) on day 3 on Collagen I. Images were taken using 10X objective on a Leica microscope.

**Figure S2**

**
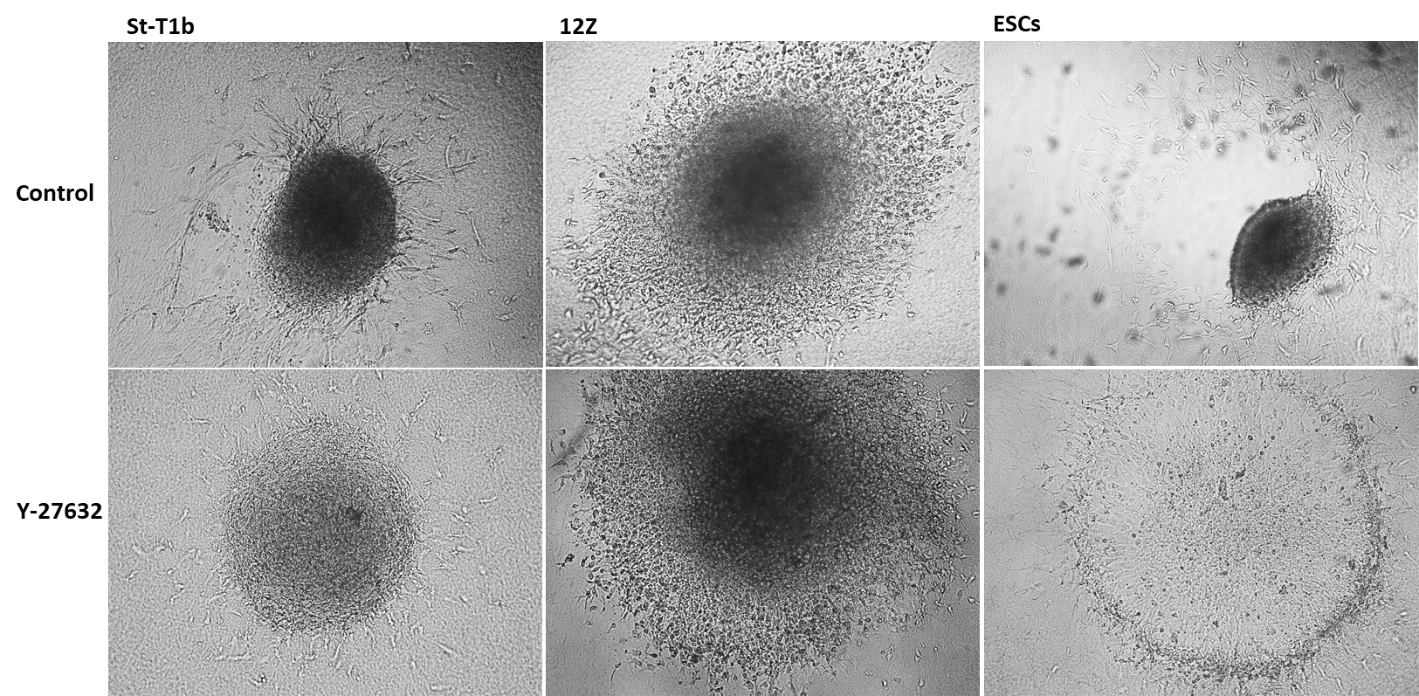
**

Figure S2. Spheroids treated with Y-27632 on day 3. The St-T1b and ESC spheroid core loses its compact structure following the treatment with Y-27632. Brightfield images obtained on a Leica microscope with 10x magnification

**Figure S3**


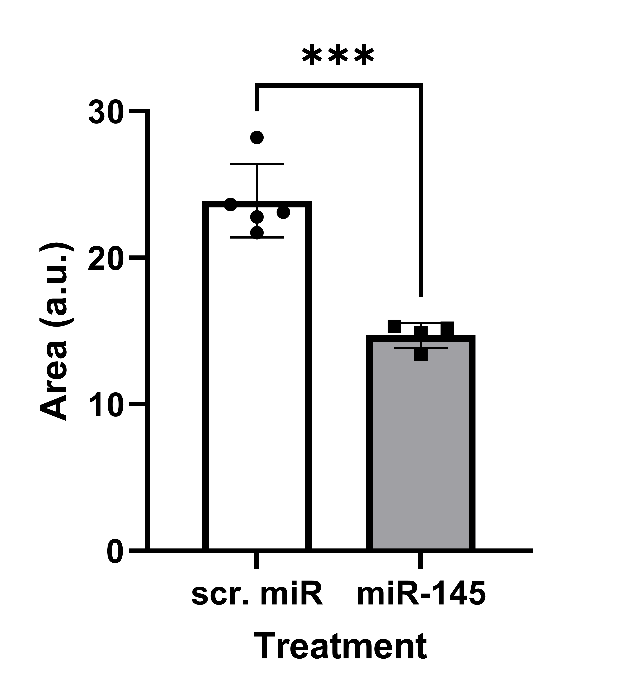


Figure S3. Area occupied by 12Z cells on Collagen I on day 3 following treatment with Scr. miR and miR-145, n=4-5 individual spheroids in 4-5 independent wells from one independent preparation. ***p<0.001, t-test

**Figure S4**

Figure S4. Effect of microRNAs on gene expression. Gene expression determined by qPCR on miR200b and scr. miR transfected 12Z spheroids. Multiple t-tests, n=3, not significant

**Supplementary Table ST1**

Table ST1. Small molecule inhibitors and microRNAs used in the study. (Q) signifies that the spheroid area/ size was quantified and (NQ)the area/size was not quantified using the measure ‘fold change in area’

|  | **Known roles** | **12Z** | **St-T1b** | **ESC** | **12Z:St-T1b** |
| --- | --- | --- | --- | --- | --- |
| NNGH | Broad-spectrum MMP inhibitor | Collagen I (Q) | Collagen I (Q) | Collagen I (Q) | Collagen I (Q) |
| Y-27632 | ROCK inhibitor  Cell survival, contractility | Matrigel (Q)  Collagen I (NQ) | Matrigel (Q)  Collagen I (NQ) | Matrigel (Q)  Collagen I (NQ) |  |
| miR-200b | EMT transition | Matrigel (Q)  Collagen I (NQ) |  |  |  |
| miR-145 | Migration and invasion | Matrigel (Q)  Collagen I (Q) |  |  |  |
